# Immune monitoring technology primer: flow and mass cytometry

**DOI:** 10.1186/s40425-015-0085-x

**Published:** 2015-09-15

**Authors:** Holden T. Maecker, Alexandre Harari

**Affiliations:** Institute for Immunity, Transplantation, and Infection, Stanford University School of Medicine, Stanford, CA USA; Centre Hospitalier Universitaire Vaudois (CHUV), Epalinges, Switzerland

## Description of the technology

**Fig. 1:**
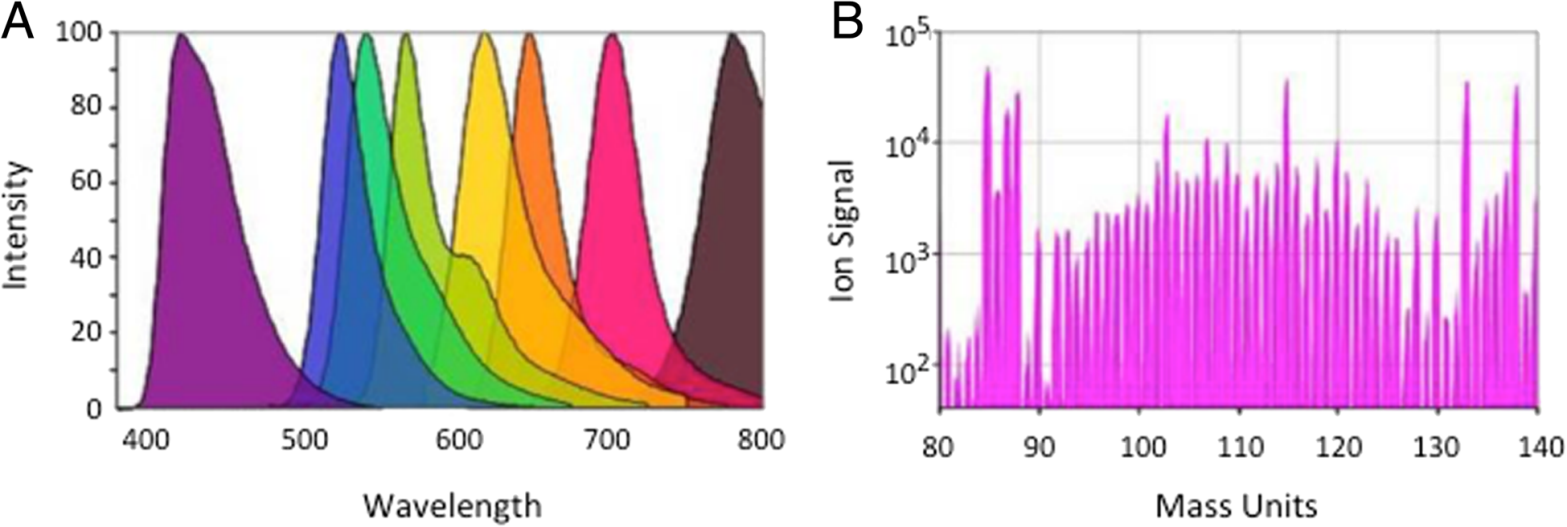
**a** Example of emission spectra of several dyes used in fluorescence flow cytometry, showing the degree of overlap and consequent spillover between detectors. **b** Ion signals detected by mass cytometry are by comparison very discrete, allowing many more simultaneous probes to be used, with little or no spillover

Flow cytometry traditionally uses fluorochrome-labeled probes, such as antibodies, to identify cells expressing the targets of those probes. A sample stream carries single cells in suspension past a laser, exciting the fluorochromes, with quantitation of the emitted fluorescent signals from each cell via optical filters and photomultiplier tubes [1]. In mass cytometry, or CyTOF, the fluorescent labels are replaced with heavy metal ions. The metal ions are chelated to a polymer, which is covalently linked to antibodies or other probes. After staining with these probes, single cells are introduced via an aerosol stream into a plasma torch, resulting in complete ionization of the labeled cells. The heavy ions are then focused via a quadrapole and enter a time-of-flight detector, where the individual ions are quantitated [2, 3]. This results in the simultaneous benefit of more available labels, with much less spillover between detector channels (Fig. 1).

## Type of data obtained/readout

Flow and mass cytometry uniquely allow for the quantitation of multiple parameters on many individual cells. Up to 17 or more parameters are possible with fluorescence, while 40 or more parameters can be quantitated with mass cytometry [4]. It is not unusual to collect data on 10^5^–10^7^ cells per sample by either technique. The data from each sample is compiled in a Flow Cytometry Standard (FCS) file, for both flow and mass cytometry. The FCS file lists the intensities obtained for each probe on each individual cell. Analysis of FCS files can be carried out in any of a number of commercial software packages, and involves sequential “gating” or selection of populations of interest. For example, single cells might first be selected based on light scatter parameters; then live cells gated by exclusion of a viability dye; then lymphocytes identified by a combination of forward and side-angle light scatter; then T cells selected by expression of CD3; etc. Note that mass cytometry does not allow for light scatter properties to be measured, so all gating is done on the basis of labeled probes, in addition to cell length (as measured by the time duration of the cell’s ion cloud). Accurate quantitation of the proportions of even rare populations of cells can be made if enough events are collected; as can relative levels of expression of cellular proteins such as activation markers, intracellular cytokines, or signaling proteins. Absolute cell counts and quantitation of molecules of bound fluorophore/metal are also possible, for example, with reference to standardized labeled beads. Automated gating algorithms are also available, including unsupervised clustering and dimension reduction techniques [5–8].

## Limitations of the approach

Pre-conjugated antibodies are now readily available for both flow and mass cytometry, though the latter generally still requires a few conjugates to be made in-house, in order to complete a specific high-parameter panel. Sensitivity to detect low-abundance proteins can be an issue for both platforms. In general, the best fluorochromes have a detection limit of about 40 molecules per cell, while the detection limit for mass cytometry is about 400–500 molecules per cell. Sensitivity is influenced by factors such as autofluorescence (in traditional flow cytometry) and non-specifically bound antibodies (in both platforms). Compensation for spectral overlap can reduce sensitivity and introduce artifacts in fluorescence flow cytometry. In both platforms, there can be loss of cells in sample preparation washing steps, although mass cytometry generally requires more washing steps than fluorescence assays. Additionally, there are cell losses in the mass cytometer itself, such that data are captured on only about 30 % of introduced cells (improved to 50 % in the latest version of CyTOF instrumentation). Collection speed is also much lower in mass cytometry (300–500 events per second, compared to several thousand events/second in fluorescence flow cytometry).

## Types of samples needed and special issues pertaining to samples

Cells for these assays need to be in single-cell suspension. Debris and cell aggregates can interfere with the running of samples and with the interpretation of data. Because of the potential for cell loss, samples with <10^5^ cells are usually not appropriate for either flow or mass cytometry. Functional assays, such as cytokine production, or analysis of phospho-signaling proteins, require cells with good viability. Overnight shipping of blood and cryopreservation of PBMC can compromise these readouts. Variability in sample handling, acquisition, and analysis are all significant in affecting results [9]. Multiple approaches can mitigate these factors, from use of lyophilized reagents [10] to “barcoding” of samples to produce a single composite sample for purposes of uniform processing and acquisition [11–13].

## Level of evidence

Flow cytometry is backed by thousands of publications and over 30 years of development. Mass cytometry is more recent, but has seen an exponential rise in publications. Several flow cytometry assays are used in FDA-approved diagnostic tests. Both platforms can produce strong evidence, provided that appropriate controls are included.

## References

[1] Dunne JF, Maecker HT, Nijkamp FP (2004). Flow cytometry. Principles of immunopharmacology F.

[2] Ornatsky O, Bandura D, Baranov V, Nitz M, Winnik MA, Tanner S (2010). Highly multiparametric analysis by mass cytometry. J Immunol Methods.

[3] Tanner SD, Baranov VI, Ornatsky OI, Bandura DR, George TC (2013). An introduction to mass cytometry: fundamentals and applications. Cancer Immunol Immunother.

[4] Bendall SC, Nolan GP, Roederer M, Chattopadhyay PK (2012). A deep profiler’s guide to cytometry. Trends Immunol.

[5] Aghaeepour N, Finak G, Hoos H, Mosmann TR, Brinkman R, Gottardo R, Scheuermann RH, FlowCAP Consortium, DREAM Consortium (2013). Critical assessment of automated flow cytometry data analysis techniques. Nat Methods.

[6] Bendall SC, Simonds EF, Qiu P, Amir EAD, Krutzik PO, Finck R, Bruggner RV, Melamed R, Trejo A, Ornatsky OI, Balderas RS, Plevritis SK, Sachs K, Pe’er D, Tanner SD, Nolan GP (2011). Single-cell mass cytometry of differential immune and drug responses across a human hematopoietic continuum. Science.

[7] Qiu P, Simonds EF, Bendall SC, Gibbs KD, Bruggner RV, Linderman MD, et al. Extracting a cellular hierarchy from high-dimensional cytometry data with SPADE. Nat Biotechnol. 2011;29:886–91. 10.1038/nbt.1991 PMC3196363 21964415

[8] Chester C, Maecker HT. Algorithmic Tools for Mining High-Dimensional Cytometry Data. The Journal of Immunology 2015;195:773–9. 10.4049/jimmunol.1500633 PMC4507289 26188071

[9] Maecker HT, McCoy JP, Nussenblatt R. Standardizing immunophenotyping for the Human Immunology Project. Nat Rev Immunol. 2012;12:191–200. 10.1038/nri3158 PMC3409649 22343568

[10] Maecker HT, Rinfret A, D’Souza P, Darden J, Roig E, Landry C, Hayes P, Birungi J, Anzala O, Garcia M, Harari A, Frank I, Baydo R, Baker M, Holbrook J, Ottinger J, Lamoreaux L, Epling CL, Sinclair E, Suni MA, Punt K, Calarota S, El-Bahi S, Alter G, Maila H, Kuta E, Cox J, Gray C, Altfeld M, Nougarede N, Boyer J, Tussey L, Tobery T, Bredt B, Roederer M, Koup R, Maino VC, Weinhold K, Pantaleo G, Gilmour J, Horton H, Sekaly RP (2005). Standardization of cytokine flow cytometry assays. BMC Immunol.

[11] Krutzik PO, Nolan GP (2006). Fluorescent cell barcoding in flow cytometry allows high-throughput drug screening and signaling profiling. Nat Methods.

[12] Zunder ER, Finck R, Behbehani GK, Amir E-AD, Krishnaswamy S, Gonzalez VD, Lorang CG, Bjornson Z, Spitzer MH, Bodenmiller B, Fantl WJ, Pe’er D, Nolan GP (2015). Palladium-based mass tag cell barcoding with a doublet-filtering scheme and single-cell deconvolution algorithm. Nat Protoc.

[13] Mei HE, Leipold MD, Schulz AR, Chester C, Maecker HT (2015). Barcoding of live human peripheral blood mononuclear cells for multiplexed mass cytometry. J Immunol.

